# Supplementary material for: Deep Proteomic Investigation of Metabolic Adaptation in Mycobacteria under Different Growth Conditions
Source: Proteomes. 2023 Dec 7;11(4):39. doi: 10.3390/proteomes11040039 (PMC10747050; doi:10.3390/proteomes11040039)
Supplement: Supplementary file 1 [file proteomes-11-00039-s001.zip › proteomes-2658962-supplementary.pdf]

Deep proteomic investigation of metabolic adaptation in mycobacteria under different growth conditions

Mariia Zmyslia [1,*], Klemens Fröhlich [2,*], Trinh Dao [1,3,4], Alexander Schmidt [2] and Claudia Jessen-Trefzer [1,4#]

[1]University of Freiburg, Faculty of Chemistry and Pharmacy, Albertstrasse 21, 79104 Freiburg, Germany

[2]Biozentrum Basel, Proteomics Core Facility, Spitalstrasse 41, 4056 Basel, Switzerland

[3]University of Freiburg, Spemann Graduate School of Biology and Medicine (SGBM), Albertstrasse 19A, 79104 Freiburg, Germany

[4]The Center for Integrative Biological Signaling Studies, University of Freiburg, 79104 Freiburg, Germany.

[*]equal contribution

[#]Correspondence: claudia.jessen-trefzer@pharmazie.uni-freiburg.de

Supporting Figures and Tables

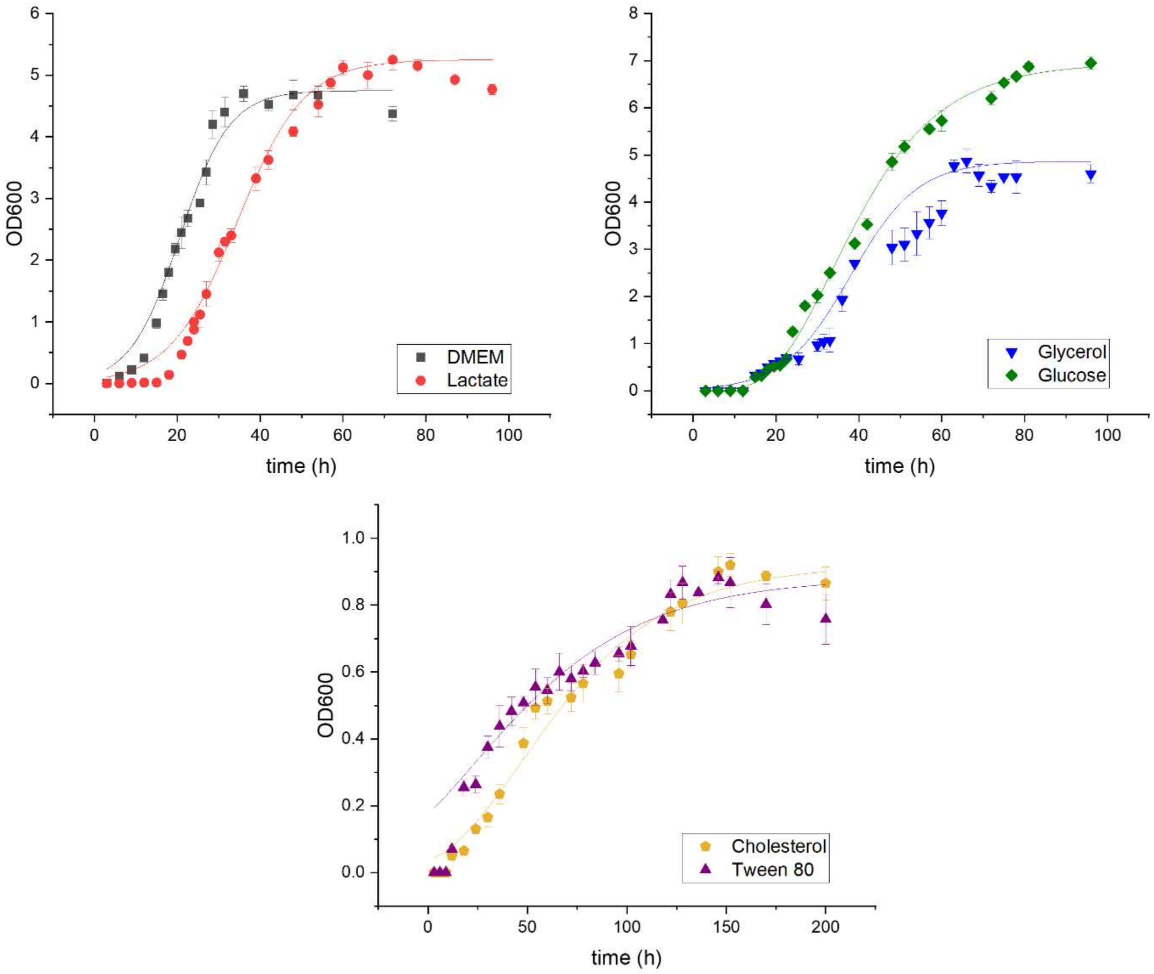

Figure S1. The growth curve analysis of *M. smegmatis* mc^{2}155 was conducted under six distinct conditions, each utilizing a specific carbon source. Growth assessments were performed in minimal Sauton's medium supplemented individually with cholesterol, D-glucose, L-lactate, glycerol, Tween 80, and in DMEM medium with 10% FCS.

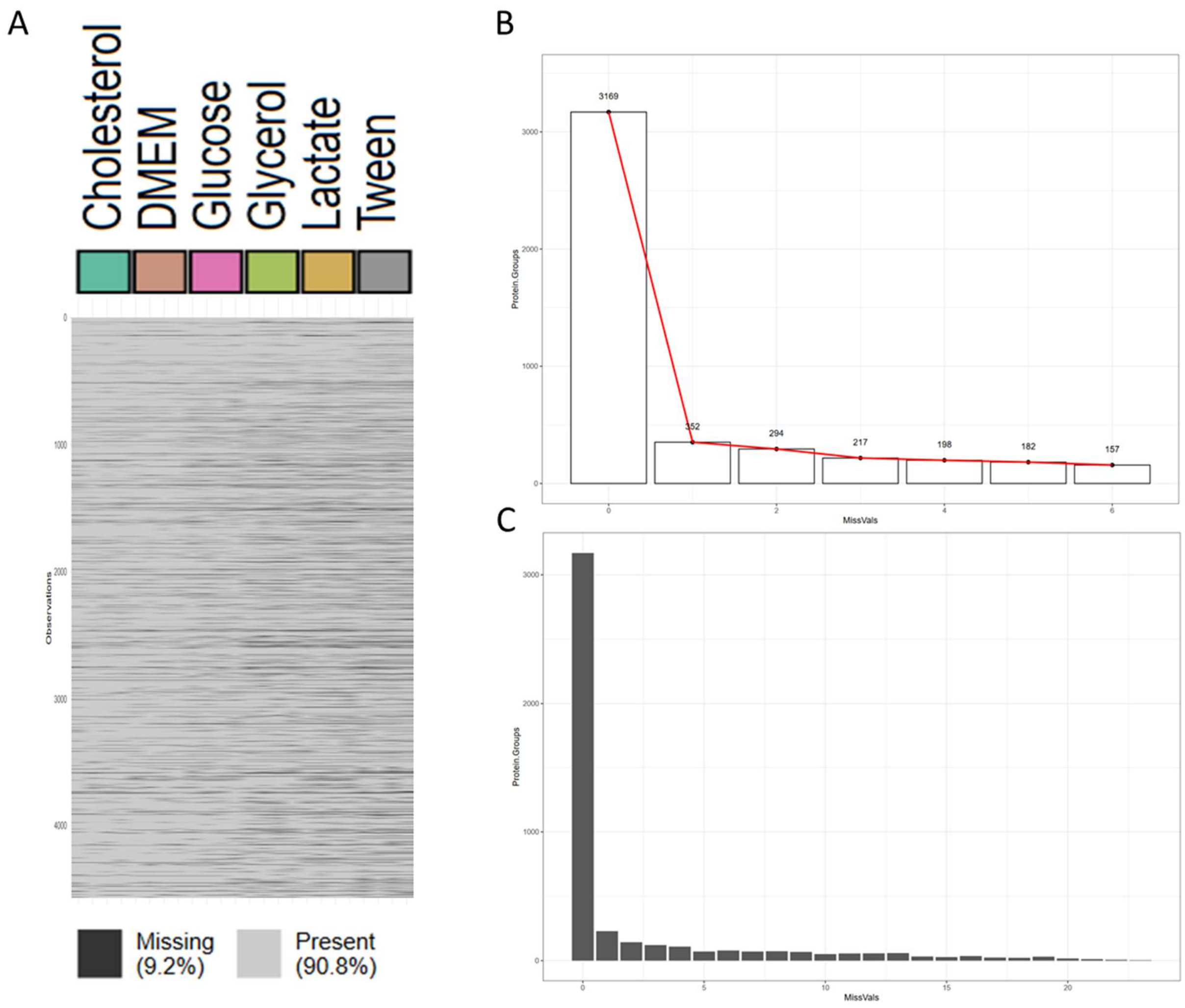

Figure S2. Visualization of Missing Values on Protein Group Level. A) Missing protein group values over replicates grouped by condition. B) Missing Protein Group Values summarized by condition. 0 means that protein groups were present in all replicates of all conditions, 6 means that protein groups had missing values in at least one replicate in all 6 conditions. C) Missing Values per protein group over all replicate. 0 means that protein groups were present in all replicates, 23 means that protein group was only present in one replicate.

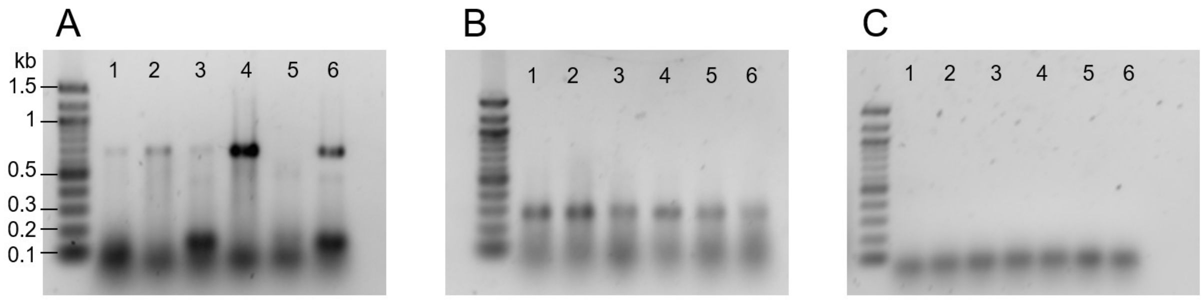

Figure S3. Agarose gel analysis of RT-PCR for MSMEI_3870 (A) and MSMEI_2690 (B) for diverse growth conditions: Tween 80 (Lane 1), Cholesterol (Lane2), D-Glucose (Lane 3), L-Lactate (Lane 4), Glycerol (Lane 5), DMEM (Lane 6). Analysis was performed in the presence (A, B) or absence of reverse transcriptase (C, representative image for the negative control using MSMEI_2690 primers). Expected PCR product size MSMEI_3870: 662 bp, MSMEI_2690: 275 bp. Respective log_{FC} under the tested growth conditions *vs* Glycerol as determined by proteomics: Tween 80 (log_{FC} 2.70), Cholesterol (log_{FC} 2.07), D-Glucose (log_{FC} 2.81), L-Lactate (log_{FC} 5.54), DMEM (1.77).

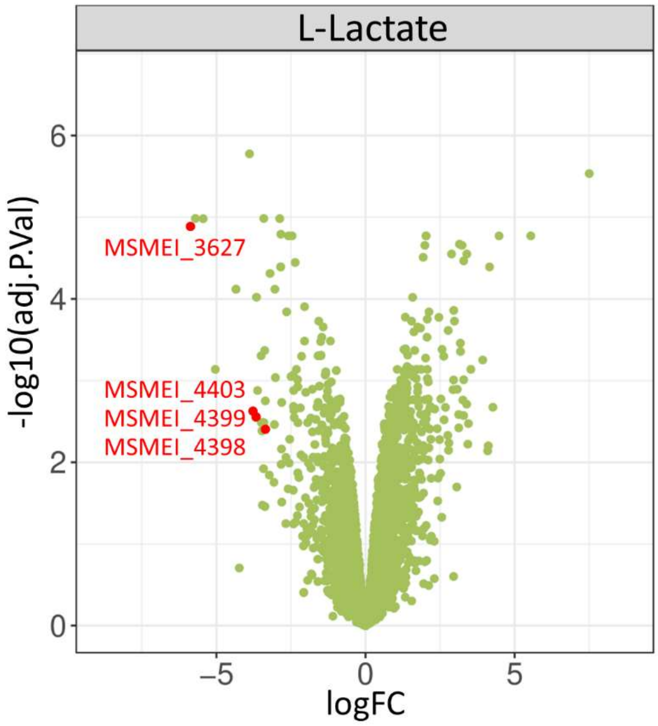

Figure S4. Volcano plot illustrating proteins with differential expression in response to the utilization of L-lactate as a carbon source for the cultivation of *M. smegmatis*, compared to the glycerol condition. Top downregulated hits (> 3-fold log_{FC}) involved in mycobactin synthesis are highlighted in red.

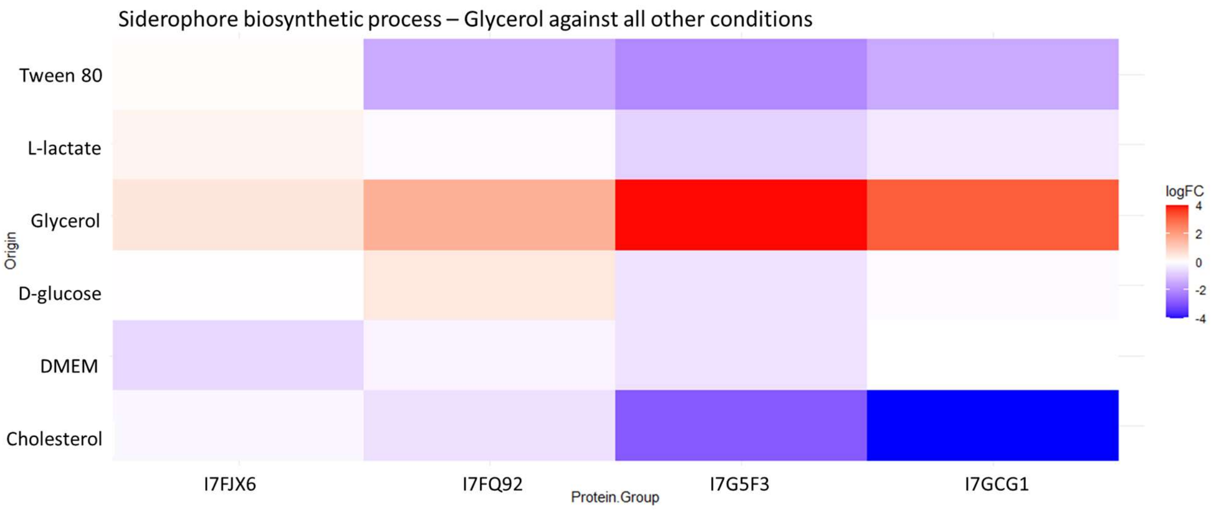

Figure S5. Heatmap of differentially expressed proteins involved in siderophore biosynthesis (glycerol condition compared to all other conditions).

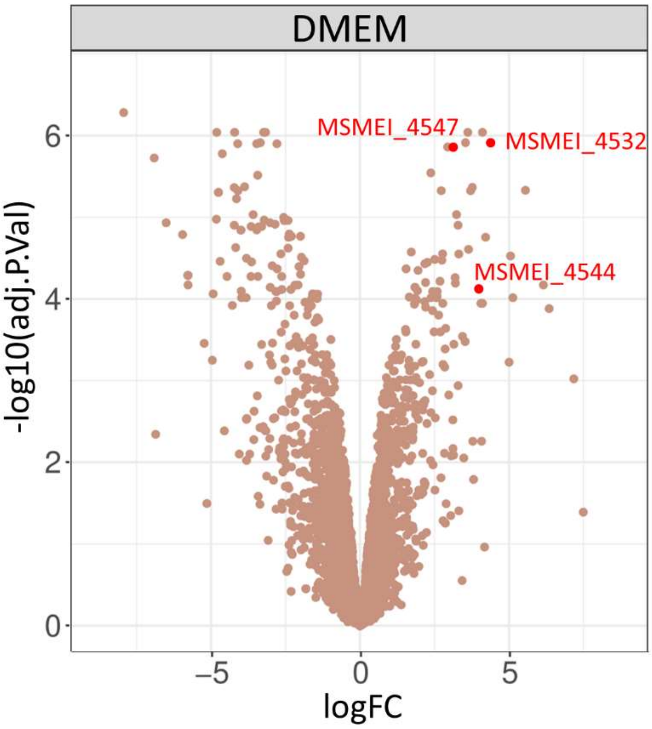

Figure S6. Volcano plot illustrating proteins with differential expression in response to the utilization of DMEM as a carbon source for the cultivation of *M. smegmatis*, compared to the glycerol condition. Top upregulated hits (> 3-fold $\log_{FC}$) involved in *myo*-inositol catabolism are highlighted in red.

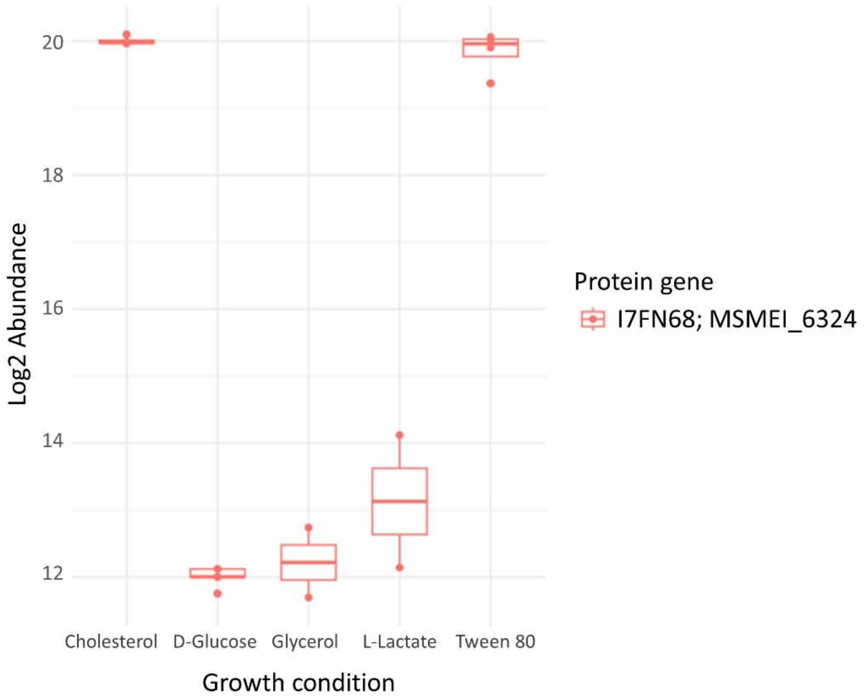

Figure S7. Boxplot of log2 protein abundance for putative membrane transporter MSMEI_6324 (I7FN68) over all conditions. Replicates shown as points.

Table S1. Primers used in this study.

MSMEI_2690 FW	CGACTACACCAAGGGCTACAAGTTC
MSMEI_2690 REV	TGGTCTGGTCCAGCGAGATG
MSMEI_3870 FW	CAGTAGTGACCGAGCGACATG
MSMEI_3870 REV	CGGCGTACTTCCAGCTGTATTAC
